# Construction of an Influenza D Virus with an Eight-Segmented Genome

**DOI:** 10.3390/v13112166

**Published:** 2021-10-27

**Authors:** Hiroho Ishida, Shin Murakami, Haruhiko Kamiki, Hiromichi Matsugo, Misa Katayama, Wataru Sekine, Kosuke Ohira, Akiko Takenaka-Uema, Taisuke Horimoto

**Affiliations:** Department of Veterinary Microbiology, Graduate School of Agricultural and Life Sciences, The University of Tokyo, Tokyo 113-8657, Japan; i.hiroho417@gmail.com (H.I.); shin-murakami@g.ecc.u-tokyo.ac.jp (S.M.); asotus2155@gmail.com (H.K.); matsugo-hiromichi@g.ecc.u-tokyo.ac.jp (H.M.); chapon5ktym@gmail.com (M.K.); wataru-sekine@g.ecc.u-tokyo.ac.jp (W.S.); kosuke-ohira@g.ecc.u-tokyo.ac.jp (K.O.); atakiko@mail.ecc.u-tokyo.ac.jp (A.T.-U.)

**Keywords:** bovine respiratory disease complex, influenza D virus, mutant, recombinant virus, reverse genetics, nonstructural protein

## Abstract

Influenza D virus (IDV) may cause the bovine respiratory disease complex, which is the most common and costly disease affecting the cattle industry. Previously, we revealed that eight segments could be actively packaged in its single virion, suggesting that IDV with the seven-segmented genome shows an agnostic genome packaging mechanism. Herein, we engineered an eight-segmented recombinant IDV in which the *NS1* or *NS2* genes were separated from NS segment into independent segments (NS1 or NS2 segments, respectively), leading to monocistronic translation of each NS protein. We constructed two plasmids: one for the viral RNA (vRNA)-synthesis of the NS1 segment with a silent mutation at the splicing acceptor site, which controls NS2 transcription in the NS segment; and another for the RNA synthesis of the NS2 segment, with deletion of the intron in the NS segment. These plasmids and six other vRNA-synthesis plasmids were used to fabricate an infectious eight-segmented IDV via reverse genetics. This system enables analysis of the functions of NS1 or NS2. We tested the requirement of the N-terminal overlapping region (NOR) in these proteins for viral infectivity. We rescued a virus with NOR-deleted NS2 protein, which displayed a growth rate equivalent to that of the eight-segmented virus with intact NS2. Thus, the NOR may not influence viral growth. In contrast, a virus with NOR-deleted NS1 protein could not be rescued. These results indicate that the eight-segmented rescue system of IDV may provide an alternative method to analyze viral proteins at the molecular level.

## 1. Introduction

Influenza D virus (IDV), a new member of the family Orthomyxoviridae, was first isolated from pigs with respiratory illness in the United States in 2011 [1,2]. Further epidemiological analyses revealed that cattle were the main host [2,3] and IDVs circulated in cattle in American [2,3,4,5,6,7], Asian [8,9,10], European [11,12,13,14], and African countries [15]. IDV infection can cause mild to moderate respiratory illnesses in cattle and has been a concern for the development of the bovine respiratory disease complex (BRDC) [5,16]. BRDC is the most common and costly disease affecting the cattle industry [17,18,19]. However, antibodies against IDVs have been found in pigs [20], sheep [21,22], goats [21,22], horses [23], dromedary camels [15,24], and humans [25,26]. These findings suggest that IDVs are distributed globally in multiple animal hosts.

Influenza A and B viruses (IAV and IBV) possess eight-segmented negative sense RNA segments (PB2, PB1, PA, HA, NP, NA, M, and NS) as genomes, whereas influenza C virus (ICV) and IDV possess seven-segmented ones (PB2, PB1, P3, HEF, NP, M, and NS) as genomes [27]. However, our previous study indicated that eight ribonucleoproteins (RNPs) are actively incorporated into a single particle of ICV and IDV [28], suggesting an undefined tolerance packaging mechanism of the viral genome.

**Figure 1:**
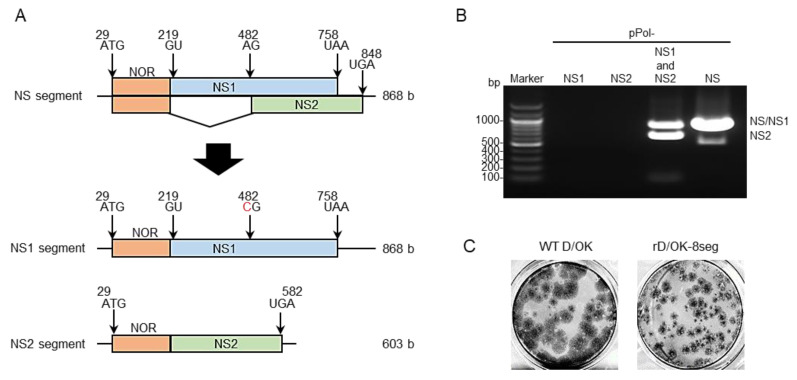
Generation of IDV with 8-segmented genome. (**A**) We created vRNA-synthesis pPol plasmids for monocistronic NS1 or NS2 segment by engineering the NS segment of D/swine/Oklahoma/1334/2011 (D/OK). N-terminal overlapping region (NOR) between NS1 and NS2 genes, NS1-, and NS2-specific regions are shown in orange, blue, and green, respectively. Each number indicates the nucleotide position and the red letter indicates the substituted nucleotide. (**B**) vRNA-synthesis pPol plasmids for NS1 and/or NS2, or NS segments were co-transfected with the other 6 (PB2, PB1, P3, HEF, NP, and M) segment vRNA-synthesis plasmids and 4 protein (PB2, PB1, P3, and NP)-expression plasmids into HEK293T cells. Thereafter, the supernatant was inoculated into ST cells. CPE was observed on ST cells following inoculation of the transfectant with pPol- D/OK-NS1 and -NS2 as well as pPol-D/OK-NS, but not with pPol- D/OK-NS1 or -NS2. Both NS1 and NS2 were detected by RT-PCR in the supernatant (rD/OK-8seg) of ST cells with 1% agarose gel electrophoresis. (**C**) Representative plaque morphologies of wild type (WT) D/OK and rD/OK-8seg in ST cells are shown by immunostaining with anti-D/OK mouse polyclonal antibody.

Similar to other types of influenza viruses, the NS segment of IDV encodes the NS1 protein, transcribed from unspliced mRNA, and the NS2 protein, transcribed from spliced mRNA [2]. The NS1 and NS2 proteins possess an overlapping identical 63-amino-acid sequence at each N-terminal region and subsequent unique sequences in the downstream region of each protein, which are translated by different reading frames, even in the C-terminal overlapped gene region (Figure 1A). According to recent reports, the IDV NS1 protein antagonizes cellular type I interferon response [29] and the NS2 protein is a nuclear export protein (NEP) that mediates the nuclear export of viral RNPs [30], similar to NS proteins of the other type viruses. However, the biological functions of these proteins have not been fully elucidated at the molecular level. As overlapping regions are present between the *NS1* and *NS2* genes, such molecular assessments may be impeded by the difficulty associated with each protein-independent mutational approach by reverse genetics.

By utilizing the possibly tolerant property of IDV genome packaging, we generated an eight-segmented recombinant virus containing independent NS1 and NS2 segments, engineered from the original NS segment, and the other six segments using reverse genetics. In addition, we demonstrated the application of this rescue system for molecular assessments of the N-terminal overlapped region of NS proteins in IDV infectivity.

## 2. Materials and Methods

### 2.1. Cells and Viruses

Human embryonic kidney 293T cells (obtained from Riken BRC, RCB2202) and swine testis (ST) cells (obtained from ATCC, CRL-1746) were maintained in Dulbecco’s modified Eagle medium (DMEM; Fujifilm Wako Pure Chemical, Osaka, Japan) supplemented with 10% fetal bovine serum (FBS) at 37 °C. D/swine/Oklahoma/1334/2011 (D/OK) (GenBank accession numbers JQ922305–JQ922311) was kindly provided by Dr. B. Hause (Kansas State University). D/OK was propagated in ST cells in Eagle’s minimum essential medium (MEM; Life Technologies/Gibco, Paisley, UK) containing 0.3% bovine serum albumin (MEM/BSA) supplemented with 0.5 µg/mL L-1-tosylamido-2-phenyl chloromethyl ketone (TPCK)-trypsin (Worthington, Lakewood, NJ, USA) and stored at −80 °C.

### 2.2. Plasmid Construction 

vRNA-synthetic plasmids (pPol-D/OK-PB2, -PB1, -P3, -HEF, -NP, -M, and -NS) containing the cDNAs of the D/OK viral genes between the human RNA polymerase I promoter and mouse RNA polymerase I terminator and eukaryotic protein expression plasmids (pCAGGS-D/OK-PB2, -PB1, -P3, and -NP) under the control of the chicken β-actin promoter were used for reverse genetics, as described previously [31,32]. A mutant pPol-D/OK-NS plasmid synthesizing only NS1 vRNA (pPol-D/OK-NS1) was constructed by introducing a synonymous mutation (A to C) at the splicing acceptor site (nucleotide (nt) position 483). A mutant pPol-D/OK-NS plasmid synthesizing only NS2 vRNA (pPol-D/OK-NS2) was constructed by deleting the intron sequence (nt positions 218–483) of the NS segment. 

A series of NS2 vRNA-synthetic plasmids with deletion mutations were prepared. To construct a plasmid with deletion of nt positions 1–78 (pPol-D/OK-NS2Δ1-26), the start codon (ATG) of the *NS2* gene was replaced with ATC, resulting in translation starting from the 27th codon (ATG), which led to the deletion of the N-terminal region at amino acid (aa) positions 1–26. We also constructed pPol-D/OK-NS2Δ27-46, Δ47-63, Δ64-87, Δ88-110, Δ111-121, Δ122-145, or Δ146-155 plasmids, in which the nucleotide sequences constituting amino acids at positions 27–46, 47–63, 64–87, 88–110, 111–121, 122–45, or 146–155 were deleted, respectively. Furthermore, the 156th codon (AAC) was replaced with a stop codon (UAG), thereby resulting in the pPol-D/OK-NS2Δ157-184 plasmid, in which the 156-184th amino acids were deleted. 

We also modified the pPol-D/OK-NS, -NS1, and -NS2 plasmids to create a mutant virus without the NOR (at aa positions 1–63) between NS1 and NS2 proteins. All ATG codons at nt positions 1–186 in these plasmids were replaced with ATC, and the 63rd codon (GAA) was replaced with ATG, constructing pPol-D/OK-NSΔNOR, -NS1ΔNOR, or -NS2ΔNOR plasmids, which led to the deletion of aa positions 1–63 of NS1 and/or NS2. 

The NS1 or NS1ΔNOR protein-expression plasmid, pCAGGS-D/OK-NS1, or -NS1ΔNOR, was prepared by PCR amplification of each coding region from pPol-D/OK-NS1 or -NS1ΔNOR using KOD FX Neo (TOYOBO, Osaka, Japan) and specific primers with 15 nucleotide-overlapping sequences to the cloning site of pCAGGS. The PCR product was cloned into EcoRI/XhoI sites using Gibson Assembly Master Mix (New England Bio Labs, Ipswich, MA, USA). 

### 2.3. Reverse Genetics 

Reverse genetics was performed as previously described [31], with some modifications. Briefly, 60% confluent HEK293T cells on a 6-well plate were transfected with 12 plasmids of different amounts (0.6 μg of each of the pPol-D/OK-PB2, -P3, and -NP, 0.1 μg of each of the pPol-D/OK-PB1, -HEF, -M, -NS1, and -NS2, 1.0 μg each of the pCAGGS-D/OK-PB2, -PB1, -P3, and -NP) using TransIT-293T (Mirus Bio, Madison, WI, USA), according to the manufacturer’s instructions. DNA and 12.8 μL of the transfection reagent were mixed and incubated at 23 °C for 20 min. The mixtures were then added to the cells and incubated at 37 °C. At 2 days post-transfection, the supernatants were removed, and cells were washed twice with Opti-MEM (GIBCO/Life Technologies Japan, Tokyo, Japan) before the addition of 2 mL of Opti-MEM containing 0.3% BSA. The cells were then incubated for an additional 5 days at 37 °C. TPCK-trypsin (0.5 μg/mL) was added to the collected culture supernatant, and the mixture was inoculated on ST cells for 1 h. The cells were washed twice with MEM before addition of 2 mL of MEM/BSA containing TPCK-trypsin (0.5 μg/mL), and incubated at 37 °C. At 5 days post-infection, supernatants were collected, and viruses were titrated in ST cells by plaque assay.

### 2.4. Plaque Assay 

The plaque assay was performed as described previously [31]. Briefly, confluent ST cells on a 12-well plate were inoculated with 0.1 mL each of 10-fold serially diluted viruses in MEM/BSA and incubated for 1 h at 37 °C. Cell were then washed with MEM/BSA, covered with 1 mL of MEM/BSA containing TPCK-trypsin (0.5 μg/mL) and 1% Seakem GTG agarose (Lonza Japan, Chiba, Japan), and incubated at 37 °C for 3 days. Subsequently, 30% formalin in PBS (0.5 mL) was added to each well to fix the cells at 4 °C overnight. After formalin and agarose were removed, the cells were washed with PBS and permeabilized with 0.1% Triton X-100 in PBS for 15 min at 23 °C. After blocking with BlockAce (KAC, Hyogo, Japan), the cells were incubated with anti-D/OK mouse immune serum for 60 min, biotinylated anti-mouse IgG antibody (#B7264, Sigma-Aldrich Japan, Tokyo, Japan) for 30 min, and then a complex with streptavidin (8 µg/mL) (Fujifilm Wako Chemicals, Miyazaki, Japan) and biotinylated peroxidase (4 µg/mL) (Invitrogen/Thermo Fisher Scientific, Tokyo, Japan) for 30 min. The plaques were visualized using a DAB peroxidase substrate kit (Vector Laboratories, Burlingame, CA, USA), according to the manufacturer’s instructions. 

### 2.5. Hemagglutination Assay 

A hemagglutination (HA) assay was performed in U-bottom 96-well microplates as previously described [33]. Briefly, 2-fold serial dilutions of the supernatants in 50 µL of PBS were mixed with 50 µL of 0.7% turkey red blood cells and incubated for 30 min at 23 °C. HA titers were determined as the reciprocal of the highest virus dilution showing complete HA. 

## 3. Results

### 3.1. Generation of Eight-Segmented IDV by Reverse Genetics 

As a constructional strategy, we sought to use a reverse genetics system [31] to generate recombinant IDV with two NS segments modified from the original NS segment, where one segment transcribes the *NS1* gene alone (namely the NS1 segment) and the other segment transcribes the *NS2* gene alone (namely NS2 segment). To construct a viral RNA (vRNA)-synthesis plasmid for the NS1 or NS2 segment, we inactivated the splicing acceptor site of NS2 pre-mRNA by introducing A to C substitution for the former and deleting the intron sequence of NS2 pre-mRNA for the latter (Figure 1A). Thereafter, we transfected HEK293T cells with these modified plasmids together with PB2, PB1, P3, HEF, NP, and M vRNA-synthesis plasmids and PB2, PB1, P3, and NP protein expression plasmids, and finally, inoculated the supernatant into swine testicle (ST) cells. Cytopathic effects (CPE) were observed in the ST cells and both NS1 and NS2 vRNAs were detected in the supernatant (Figure 1B), which formed plaques (Figure 1C). These findings suggest the successful generation of a virus possessing eight-segmented RNAs (namely rD/OK-8seg) (Table 1). 

### 3.2. Evaluation of vRNA Content in rD/OK-8seg Virions 

**Figure 2:**
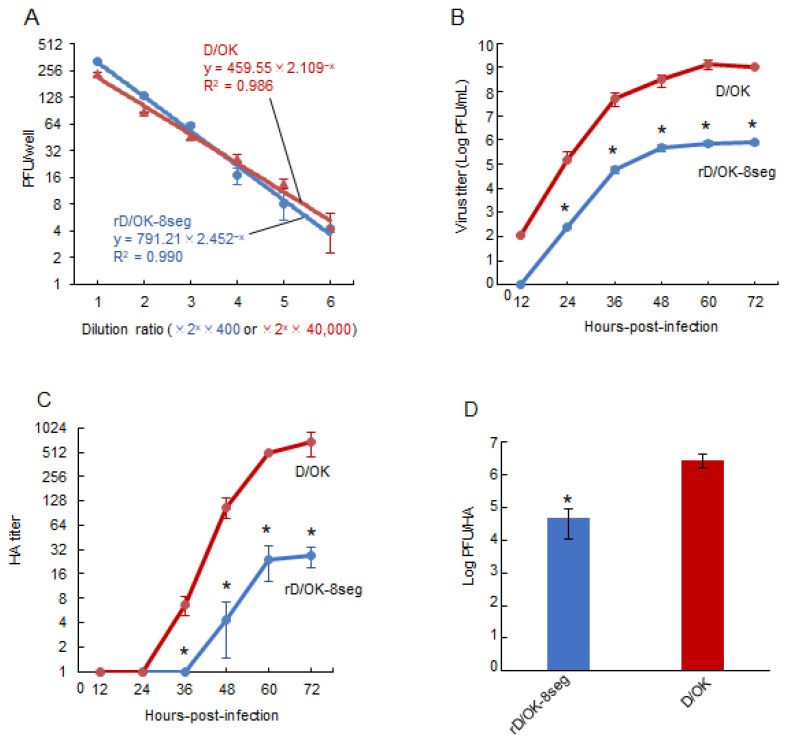
Growth properties of rD/OK-8seg. (**A**) We evaluated the correlation between dilution ratio and number of plaques in the diluents of rD/OK-8seg or D/OK, where two-fold dilutions from 400-fold for the former or 40,000-fold for the latter, respectively, were employed. Plaque numbers at each dilution are expressed as the mean titer with standard deviations (*n* = 3). The coefficient of determination (R2) was determined by regression analysis for each virus. Growth kinetics of rD/OK-8seg and D/OK was examined in ST cells (MOI of 0.0001). Viral titers were determined at 12 h intervals post-infection by (**B**) plaque assay or (**C**) HA assay and reported as the mean titer with standard deviations (*n* = 3). ANOVA (a linear mixed model) indicated the significance of the differences (*p* < 0.01) between growth kinetics of rD/OK-8seg versus D/OK by each assay. The asterisks indicate significant differences at each time-point (*p* < 0.05 by Holm test as a post hoc test). (**D**) PFU/HA ratios were determined in rD/OK-8seg and D/OK stocks in ST cells at 60 h post-infection (MOI of 0.0001) and reported as the mean titer with standard deviations (*n* = 3). The significance of the differences (*p* < 0.05) between two viruses was evaluated by the two-tailed Student-*t* test.

To confirm that the rD/OK-8seg had one set of eight vRNA segments in one particle, we serially diluted the virus stock and determined the infectious titers using the plaque assay (Figure 2A). The number of plaques with rD/OK-8seg decreased at a rate proportional to the dilution, similar to that of the wild-type D/OK, suggesting that the rD/OK-8seg packages eight RNA segments, including the NS1 and NS2 segments in a single particle. 

To confirm the possibility that infectivity of the rD/OK-8seg was induced by co-infection with two or more non-infectious viruses possessing incomplete genome sets, we independently generated seven-segmented recombinant viruses either with NS1 or NS2 segments by reverse genetics under the same rescue conditions, and co-infected the cells with these two non-infectious viruses. Although infectious viruses could be detected, a markedly lower titer of 1.0 × 10^2^ PFU/mL was found compared to that of rD/OK-8seg (7.5 × 10^5^ PFU/mL). These findings indicated that almost all infectious viruses of the rD/OK-8seg were eight-segmented and contained both the NS1 and NS2 segments. 

### 3.3. Replication Properties of rD/OK-8seg 

We assessed the growth kinetics of rD/OK-8seg in ST cells. The peak titer of rD/OK-8seg was approximately 10^3^-fold lower than that of wild-type D/OK (Figure 2B), a finding supported by its smaller plaque size (Figure 1C). To determine the cause of this discrepancy, we compared the ratios of peak infectivity titer (PFU) to HA titer (Figure 2C) between rD/OK-8seg and wild-type D/OK, which indicated a significantly lower ratio for the former (Figure 2D). These results suggest that the rD/OK-8seg stock contains a higher population of non-infectious particles than wild-type D/OK.

### 3.4. Generation of rD/OK-8seg with Deletion of N-terminal Overlapped Region of NS 

**Figure 3:**
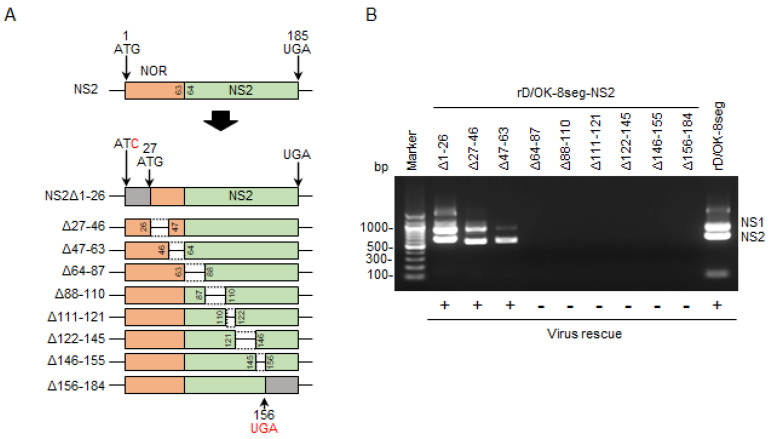

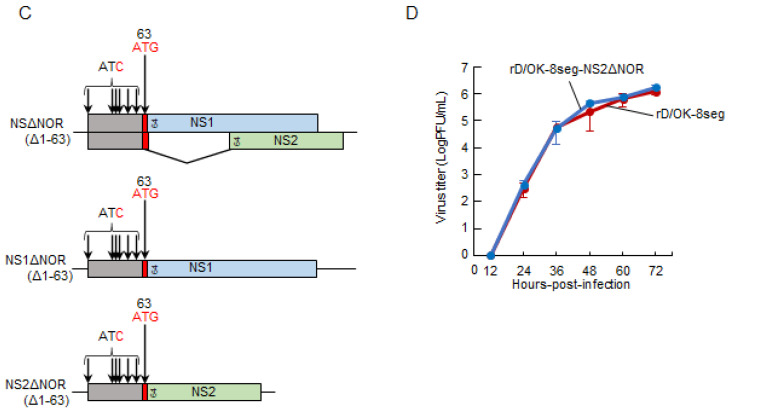
Generation of the rD/OK-8seg mutants with NS partial deletion. (**A**) We created a series of vRNA-synthesis pPol plasmids for the NS2 segment with partial deletions. NOR and specific regions of NS2 are shown in orange and green, respectively. Untranslated regions modified with a start or stop codons are shown in gray. Each number indicates the codon position from the first ATG, and the red letters indicate the substituted nucleotides. Dashed lines indicate the deleted regions. (**B**) vRNA-synthesis plasmids for each NS2 segment with partial deletion were co-transfected with the other 7 (PB2, PB1, P3, HEF, NP, M, and NS1) segment vRNA-synthesis plasmids and 4 protein (PB2, PB1, P3, and NP)-expression plasmids into HEK293T cells. Thereafter, the supernatant was inoculated into ST cells. CPEs were observed on ST cells following inoculation of the transfectant with pPol-NS2Δ1-26, Δ27-46, or Δ47-63, but not with the other 6 plasmids with deletions. Both NS1 and NS2 segments were detected by RT-PCR in the supernatants of ST cells showing CPEs with 1% agarose gel electrophoresis. The results of virus rescue were indicated at the bottom of the figure: rescued (+) or not rescued (−). (**C**) We created NOR-deleted NS, NS1, or NS2 segment by introducing mutations at all ATG codons within NOR and adding a start codon (ATG) at the 63rd position. Untranslated region by this modification, NS1-, and NS2-specific regions are shown in gray, blue, and green, respectively. Inserted start codon and the substituted nucleotides are shown in red. Each number indicates the position of the codon. The 8-segmented rescue system indicated that only rD/OK-8seg-NS2ΔNOR could be rescued. (**D**) Growth kinetics of rD/OK-8seg-NS2ΔNOR and rD/OK-8seg was assessed in ST cells (MOI of 0.0001). Viral titers were determined at 12 h intervals post-infection by the plaque assay and reported as the mean titer with standard deviations (*n* = 3). ANOVA (a linear mixed model) indicated no significant differences in growth kinetics between the two viruses.

The eight-segmented rescue system containing the NS1 and NS2 segments could independently introduce mutations into the *NS1* and/or *NS2* genes. Accordingly, we generated a series of NS2 partial deletion mutants using this system (Figure 3A). In this trial, three NS2 deletion mutants, NS2Δ1-26, Δ27-46, and Δ47-63, were generated, as confirmed by RT-PCR detection of both the NS1 and NS2 segments in the rescued viruses (Figure 3B), which indicated that these deleted regions were not required for viral growth. Interestingly, as all regions were included in the N-terminal overlapped region of the NS1 and NS2 proteins (NOR: amino acid positions 1–63), we proceeded to generate the NS2 mutant that lacked the entire NOR (Figure 3C). rD/OK-8seg-NS2ΔNOR was successfully rescued (Table 1) and its growth rate was found to be equivalent to that of rD/OK-8seg (Figure 3D).

We attempted to generate an NOR deletion NS1 mutant (rD/OK-8seg-NS1ΔNOR) eight-segment system, as well as the NOR deletion NS mutant (rD/OK-8seg-NSΔNOR) using standard reverse genetics (Figure 3C); however, these mutants were not rescued (Table 1). Such findings imply that NOR plays an essential role in the NS1 function required for IDV infectivity, but not in the NS2 function. 

## 4. Discussion

Here, we used reverse genetics to artificially generate replication-competent eight-segmented IDV containing engineered monocistronic NS1 and NS2 segments. Mutational analysis using this system revealed that the NOR of NS proteins was vital for the function of NS1 but not NS2 in viral infectivity. The eight-segmented rescue system may provide an alternative approach by serving as a useful tool for the molecular assessment of IDV infectivity. 

Although random and selective models for genome packaging mechanisms have been proposed for IAV [34], the latter is preferred according to previous studies that revealed the presence of segment-specific packaging signals at the 5′ and 3′ ends of each RNA segment [35,36,37,38,39,40,41] and the incorporation of eight different types of RNA segments into a single virus particle [42,43,44,45]. In contrast to IAV, the genome packaging mechanism of IDV, which is a newly classified influenza virus [46], remains largely unknown. As IDV adopts a selective genome packaging mechanism, it has seven different RNA segments for the virus to be infectious. However, electron microscopic analysis revealed that IDV incorporates eight RNPs in a unique “1 + 7” pattern, similar to IAVs, in which seven RNPs surround the central RNP [28]. However, the specific RNA content in the particles is unknown. IDV particles may thus possess the spatial capacity to accommodate eight RNA segments in a single particle. This postulation is supported by the fact that a ribosomal RNA lacking a specific packaging signal was incorporated as the eighth segment in artificial seven-segmented IAV particles [44]. In the present study, we generated a recombinant IDV with eight RNA segments, including the NS1 and NS2 segments, in one particle. Although packaging signal sequences were not determined for the IDV NS segment, a long range of nucleotide sequences are identical at both ends between the NS1 and NS2 segments, suggesting that two different segments with the same packaging signals may be incorporated simultaneously into one particle. Accordingly, it is hypothesized that one segment is selectively while the other is randomly incorporated, which is supported by our finding that rD/OK-8seg stock, where all eight segments must be incorporated to be infectious, may contain a larger number of noninfectious particles than rD/OK stock, where any segment is acceptable as the eighth segment, possibly leading to a lower virus titer of rD/OK-8seg stock. Collectively, these findings may provide a notion for the genome packaging mechanism of IDV, where seven different segments are selectively incorporated via packaging signals and the eighth (or more) additional segment is randomly incorporated into the particle, potentially owing to the presence of a special capacity in the particle. This notion may explain the robust establishment of our eight-segmented rescue system in this study. Further studies are needed to elucidate the genomic packaging mechanism of IDV, including the identification of packaging signal sequences in each segment and analysis of a competitive relationship among segments. 

NS1- or NS2-specific mutants could serve as useful tools for analyzing the biological functions of each NS protein on viral growth, including their interactions with other viral proteins or genomes. However, such intended mutants cannot be generated by standard reverse genetics because of the presence of overlapping regions between two *NS* genes. Alternatively, protein expression experiments have limitations in understanding the comprehensive functions of NS proteins. To circumvent this experimental pitfall, the rD/OK-8seg possessing independent NS1 and NS2 segments could be used, which allows us to generate viruses with independent mutations in NS1 or NS2. The present study provides an experimental example of the usefulness of the eight-segmented rescue system for this purpose. Using this system, we analyzed the biological necessity of NOR of NS proteins in viral infectivity. This eight-segmented system is expected to be applied to analyze the biological functions of the overlapped region between M1 and M2 proteins, which are encoded in a single segment. 

Rescue experiments with NS2-deletion mutants suggested that the NOR of NS2 contains no functional domain responsible for viral infectivity, and deletion mutants with other regions in the C-terminal region could not be generated. A recent report indicated that IDV NS2 includes three nuclear export signals (NESs) at positions 66–75, 97–107, and 136–146, which may mediate the chromosome region maintenance 1 (CRM1)-dependent nuclear export pathway, similar to IAV NS2 [30]. Our rescue experiments suggest that NS2ΔNOR, which retains these NESs, might play an equivalent role to intact NS2 in viral infectivity. In contrast, NS1 NOR (also NS NOR)-deletion mutants could not be rescued, suggesting that the NS1 NOR plays a role in viral infectivity, unlike NS2 NOR. It is well established that IAV NS1 is a multifunctional protein that mainly counteracts host antiviral defense, including interferon system [47,48,49,50], although the functionality of its NOR is unknown. A recent study showed a recombinant IAV expressing IDV NS1 rescued with low replicability in human A549 cells, indicating the difference between two NS1s [29]. Our eight-segmented rescue system will help to elucidate the biological function of NS1 NOR for IDV infectivity in future studies. 

To control BRDC, combination vaccines consisting of several respiratory viral and bacterial agents have been utilized; however, their efficacies are limited [17,18,19]. One possible reason for this limitation is the recent development of BRDCs, owing to agents that are not included in current vaccines. Indeed, metagenomic analyses have revealed a positive correlation between BRDC and IDV infection [5,16]. Therefore, the development of an IDV vaccine and its inclusion in current vaccines may help to better control BRDC. Furthermore, a possible attenuated phenotype of the rD/OK-8seg may lead to a new conceptional strategy for the development of a live vaccine for IDV, which would not revert to the wild-type phenotype in the context of genome constitution.

## Figures and Tables

**Table 1:** Generation of the rD/OK-8seg and its mutant.

Virus Segments	Number of Segments	Virus Rescue	Designation ^1^
PB2, PB1, PA, HEF, NP, M plus			
NS	7	+ ^2^	rD/OK-WT
NS1	7	−	
NS2	7	−	
NS1, NS2	8	+	rD/OK-8seg
NSΔNOR	7	−	
NS1ΔNOR, NS2	8	−	
NS1, NS2ΔNOR	8	+	rD/OK-8seg-NS2ΔNOR
NS1ΔNOR, NS2ΔNOR	8	−	

^1^ Only rescued viruses are designated. ^2^ Virus was rescued (+) or not rescued (−).

## Data Availability

Not applicable.
